# Psychometric qualities of the HLS-EU-Q16 instrument for parental health literacy in Swedish multicultural settings

**DOI:** 10.1186/s12889-021-12346-8

**Published:** 2022-02-12

**Authors:** Kirsi Tiitinen Mekhail, Bo Burström, Anneli Marttila, Josefin Wångdahl, Lene Lindberg

**Affiliations:** 1 Department of Global Public Health, K9, Karolinska Institute, 171 77 Stockholm, Sweden; 2 Centre for Epidemiology and Community Medicine, Stockholm County Council, 104 31 Stockholm, Sweden; 3 Department of Public Health and Caring Sciences, Social Medicine, BMC, Uppsala University, 751 22 Uppsala, Sweden

**Keywords:** Health literacy, HLS-EU-Q16, Parents, Migrant, Multicultural, Psychometrics

## Abstract

**Background:**

Health literacy (HL) is important for individuals in terms of knowledge and competence to make decisions about healthcare, health promotion and disease prevention. Migrants generally demonstrate lower HL levels compared to the majority populations. HL interventions among migrants are rarely studied. Thus, there is a need to find useful HL measurements for multicultural settings. The importance of understanding parents’ HL is related to their key role in providing and promoting the health of their children. This study aimed to add knowledge about the psychometric properties of the HLS-EU-Q16 instrument (Swedish version) among parents in Swedish multicultural settings.

**Methods:**

A cross sectional design was used. Totally 193 first-time parents (*N* = 193) were recruited through two child healthcare centres in Stockholm. Parents were interviewed when their infants were < 2 months old using structured questionnaires including HLS-EU-Q16. For psychometric evaluation of HLS-EU-Q16 instrument, exploratory factor analyses (EFA) were used to test internal consistency (*N* = 164). HL levels in sub-groups were explored with Kruskal-Wallis/Chi2 tests. Participants’ comments on HLS-EU-Q16 questionnaire were viewed to explore how the questions were perceived by the target population.

**Results:**

One factor solution of EFA explained 37.3% of the total variance in HLS-EU-Q16. Statistically significant differences in HL levels were found in relation to migration including language difficulties and level of education of the study population and access to support in line with previous research. Challenges related to understanding HLS-EU-Q16 questionnaire were found among participants with migrant background.

**Conclusions:**

The Swedish version of HLS-EU-Q16 could be used together with other instruments for measuring overall HL in multicultural settings. HLS-EU-Q16 appears to discriminate between different levels of HL in relation to migrant background and shorter education and limited access to support. However, other measures of HL which should be adapted to use in multicultural settings, need to be explored in further studies of parental HL and its relationship to child health in multicultural settings.

**Trial registration:**

The study was retrospectively registered (18 February 2020) in the ISRCTN registry (ISRCTN10336603).

**Supplementary Information:**

The online version contains supplementary material available at 10.1186/s12889-021-12346-8.

## Background

Health literacy is about the complex demands of modern society that require its citizens to have certain knowledge and skills in order to access health care, health promotion and disease prevention [1]. From the many different definitions of HL in the literature [2], a conceptual model by the European Health Literacy Consortium, that incorporates components from 17 different definitions of HL and aims at integrating medical and public health views of HL, was used in this study to define comprehensive HL [3]: *Health literacy is linked to literacy and entails people’s knowledge, motivation and competences to access, understand, appraise and apply health information in order to make judgement and take decisions in everyday life concerning health care, disease prevention and health promotion to maintain or improve quality of life during the life course.*

There are a number of different tools that measure HL that focus on multiple aspects of HL [4, 5]. The European Health Literacy Survey Questionnaire (HLS-EU-Q) and its various versions (HLS-EU-Q86/HLS-EU-Q47/HLS-EU-Q16/HLS-EU-Q6) [5] is based on a comprehensive definition of HL and measures the comprehensive HL of individuals in different populations [3]. The purpose of designing HLS-EU-Q was to create a tool that is both multidimensional, multinational, interdisciplinary and comprehensive, suited to measuring HL in different populations [5]. The development process of HLS-EU-Q included item development, pre-testing, field testing, external consultation, plain language check, and translation from English into other languages [5]. HLS-EU-Q originally included 47 items [5] while HLS-EU-Q16 is recommended when a shorter HL measurement measure of HL is required [6]. HLS-EU-Q16 (in English) is a constructed and validated short version of HLS-EU-Q47. However, as a short version it has a more limited conceptual representation [6, 7].

Individuals with adequate HL gain from an improved self-perceived health status, better health knowledge, shorter periods of hospitalisation and less frequent use of different healthcare services and lower health care costs [3]. However, low levels of HL increase health care costs on a societal level [8] and affect individual’s ability to understand practical health issues in their everyday life [9], such as using the appropriate medication or correctly interpreting medical labels and the different kinds of health messages [10].

According to previous research, educational achievement is the most important determinant of HL [11, 12]. Significantly higher HL has been seen in individuals with higher level of education [1, 8]. Access to social support is regarded as an important factor in the association between HL and general health [13]. Social networks can provide individuals with health-related support and the HL scores of individuals and their family members may be positively correlated [14].

Migrants are a group that generally have a lower HL compared to the majority populations [8]. Underlying causes are economic, social and language barriers, resulting in poorer access to and less use of information about health promotion, disease prevention and healthcare services [8]. Apart from poor language proficiency, lower educational level, gender, higher age, and poverty may impact HL negatively, with the exception of migrants in healthcare professions, who demonstrate higher levels of HL [15]. Refugees living in Sweden have also shown limited HL levels. In a recent study, only 20% of refugees demonstrated an adequate level of comprehensive HL [16]. Another Swedish study showed that refugees with low HL reported poor health and/or that they had refrained from seeking health care due to language barriers, a belief that they would not receive the required help, long waiting times or not knowing how to access health care [17].

Parents’ HL, their ability to access, understand, appraise, apply health-related information and act on it affect their children’s health in many ways, as they are in a key role in providing and promoting the health of their children. To our knowledge, research about migrant parents’ HL and efforts to develop HL interventions among this group are limited. For this reason, it is important to have a validated instrument for measuring HL among migrants and in the evaluations of interventions that aim to improve HL in migrant populations. The version of HLS-EU-Q that is available in Swedish [18], which has been used in this study to measure the comprehensive HL of first-time parents in multicultural settings, has not been validated in Sweden.

This study aimed to gain knowledge about the psychometric properties of HLS-EU-Q16 (Swedish version) used among first-time parents living in Swedish multicultural settings.

## Method

### Design

This study has been included in the evaluation of an extended postnatal home visiting programme in Stockholm, Sweden. The programme design has been reported elsewhere [19]. To analyse the aims of this study, we used a cross-sectional design.

All methods were performed in accordance with the Declaration of Helsinki, research involving human participants. Ethical approval for this study was granted by the Stockholm Regional Ethical Review Board (registration number 2017/1587-31/5).

### Setting

The two study settings are classified as socioeconomically disadvantaged areas, characterised by higher unemployment, a higher proportion of the population with a lower level of education and poorer health compared to the average population in Stockholm County [20]. In the study settings, around 60–90% of the populations are from migrant backgrounds [21]. The word ‘migrant’ has multiple definitions [22]. However, in this study, ‘migrant’ has been used to describe participants born outside Sweden and ‘multicultural setting’ has been used to describe the study population that includes both participants born in Sweden and in several other countries.

### Participants

A total of 212 eligible families with their first child were invited to participate in the study, of which 155 (73.1%) chose to participate, resulting in a total sample size of parents *N* = 193. During the interviews, both parents were interviewed at the same time in 76 (39.4%) of cases, only mothers in 105 (54.4%) of cases and only fathers in 12 (6.2%) of cases. When both parents were present and interviewed at the same time, they were actively encouraged to give their own individual responses regarding HLS-EU-Q16 and the whole questionnaire used for the interview. Their responses were written down in two separate questionnaires.

### Measures

Comprehensive HL was measured by using HLS-EU-Q 16 (Swedish version) [18] as part of a longer structured questionnaire. The Swedish version of HLS-EU-Q16 [18] has been translated from the validated English questionnaire HLS-EU-Q16 [5]. Existing translations of HLS-EU-Q16 into different languages (Arabic, Dari, Farsi, Somali and Sorani) were also available as a support to interpreters. Each question of HLS-EU-Q16 was answered by choosing one response of the available choices: ‘very difficult’, ‘fairly difficult’, ‘fairly easy’ and ‘very easy’ [7]. The original version of HLS-EU-Q16 measures HL in the three domains of health care (seven items), disease prevention (five items) and health promotion (four items) [7].

For the psychometric analyses of HLS-EU-Q16, each of the 16 questions were coded according to the following: ‘very difficult’ = 1 point, ‘fairly difficult’ = 2 points, ‘fairly easy’ = 3 points or ‘very easy’ = 4 points, giving a total of 16–64 scores [7]. The HLS-EU-Q16 manual recommends dichotomising the answers from HLS-EU-Q16 (‘very difficult’/‘fairly difficult’ = 0, ‘fairly easy’/‘very easy’ = 1; total 0–16 scores) and dividing the total scores into three categories of HL (‘likely inadequate HL ‘(0–8 scores), ‘likely problematic HL’ (9–12 scores) and ‘likely sufficient HL’ (13–16 scores) [7], which in this study was merely used for the pre-analysis of HL.

The sociodemographic questions in the study covered parental age, country of birth, approximate period of residence in Sweden, need for an interpreter, marital status, and level of education. The participants were also asked how they assessed their own and their child’s health on a scale of 1–5 (‘very poor’ = 1, ‘poor’ = 2, ‘average’ = 3, ‘good’ = 4 and ‘excellent’ = 5). Furthermore, participants were asked about their access to support from their social networks when required on a scale of 1–3 (‘never/mostly not’ = 1,‘mostly of the time’ = 2 and ‘always’ = 3) and stating their main source of income prior to the birth of their child.

### Procedure

Parents who registered their first child in the two different child healthcare (CHC) centres in the study settings from October 2017 to April 2019 and who consented to be interviewed were included in the study when their infant was < 2 months. In the recruitment process, CHC nurses provided brief information to the potential participants about the ongoing study. Parents who were interested in receiving further information about the study were provided with written and oral information about the aim of the study, mainly by the first author (KTM). Written informed consent was obtained at the interviews. Information about the study and informed consent were available in Swedish, English, Arabic and Somali and interpreters helped the participants to understand the information and the contents of the informed consent, when necessary. The parents themselves could decide whether one or both would participate.

Questionnaire-based structured interviews were conducted mainly face to face (*n* = 190) at the CHC centres. Responses of the participants were noted in the questionnaires. Three out of 193 interviews were conducted by phone as the parents preferred phone calls. Face-to-face interviews, rather than self-completed questionnaires, were chosen to capture the parents’ comments on the HLS-EU-Q16 items. Furthermore, previous research from one of the study sites showed a relatively high participation rate through recruitment at CHC centres for face-to-face interviews [23]. Using interpreters was another reason for conducting interviews rather than using self-completed questionnaires. Parents could choose whether they wanted the interviews to be conducted in Swedish, English or use an interpreter. Interpreters were present in 43 (22.3%) of the interviews. In five cases, a relative/friend functioned as an interpreter. Thirteen different languages were used in the interviews. Common languages used after Swedish (118 interviews; 61.1%) were English (19 interviews; 9.8%) and Arabic (19 interviews; 9.8%). Five of the interviews were conducted using both English and Swedish. The duration of the interviews that were measured (*n* = 23) was 11–30 min.

### Statistical analyses

Statistical and descriptive data were analysed using SPSS Statistics, Version 26. Data analyses were started on with set of data including 193 participants.

### Missing values

Initially three participants with more than two incomplete responses in the HLS-EU-Q16 items were excluded from the analyses according to the HLS-EU-Q16 manual [7]. Incomplete responses from HLS-EU-Q16 items (*N* = 29 (9.5%)) were treated as missing values and replaced in the analyses by series means as the percentage of missing values for each item in HLS-EU-Q16 was < 5% (Additional file 1: Table 1).

### Univariate and multivariate normality and outliers

Univariate statistics for the items in HLS-EU-Q16 (*N* = 190), including screening for missing values are displayed in Additional file 1: Table 1. Test of normality (Kolmogorov-Smirnov) showed that data was not normally distributed, detailed results are reported in Additional file 1: Table 2. None of the items indicated skewness in the distribution (−.631-.101) (Additional file 1: Table 2). Kurtosis was absent (−.393 - .783), except of for one item that showed a tendency to be right skewed (2.049) (Additional file 1: Table 2).

**Fig. 1:**
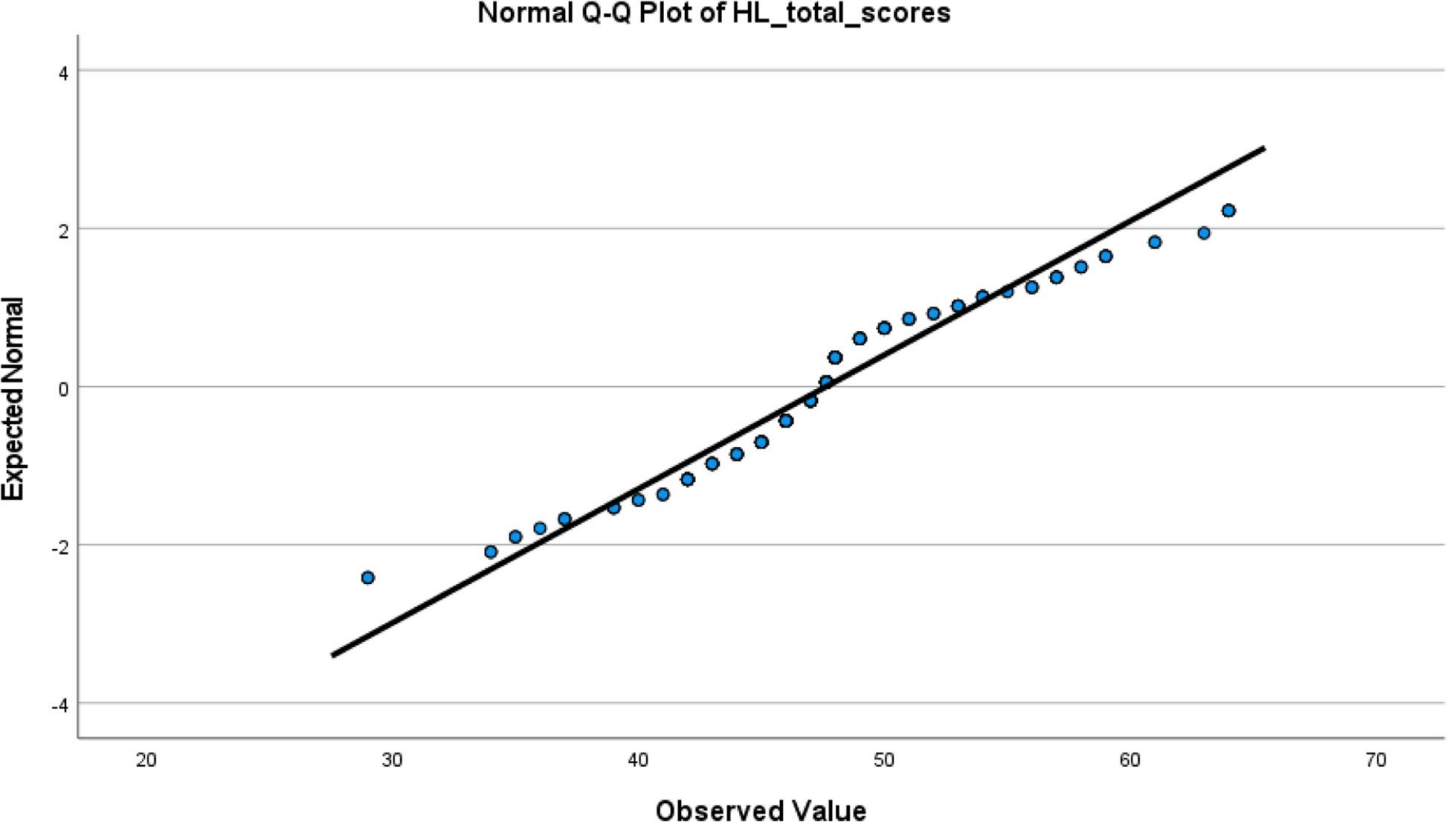
Normal Q-Q plot of total HLS-EU-Q16 scores

Regarding multivariate normality, z-kurtosis and z-skewness should be < 5 [24] and the *p*-value ≥0.05 for normality. In our set of data z-kurtosis was > 5 and p-value < 0.05 tested by Mardia’s multivariate normality test which indicated non-normal distribution (Additional file 1: Table 3). Normal Q-Q plot of total HLS-EU-Q16 scores did not show a straight line of the normal distribution [25] (Fig. 1). Conducting multivariate outliers test in SPSS with Mahalanobis distances, identified 26 multivariate outliers through p-value of Chi-square distribution (*p* < .001) [26].

### Sample size in the analyses

Removal of multivariate outliers led to sample size *N* = 164 that was used for the analyses of this study. Analyses based sample size *N* = 190 (without removal of the multivariate outliers) are reported in Additional file 2.

No significant differences were found in background variables (age, region of birth, time in Sweden, need of interpreter and education) or other variables (access to support, parental health and child health) of those participants (*N* = 26) that were removed from the data set compared to those who were included in the analyses (*N* = 164), tested by chi-square and Kruskal-Wallis tests.

### Psychometric properties

Pre-analyses (*N* = 190), based on the dichotomisation of the HLS-EU-Q16 scores [7] showed that the majority, more than 60% of the participants, would end up in the category of ‘likely sufficient HL’. For this reason, the analyses of psychometric properties were based on the sum of the total HLS-EU-Q16 scores, giving a wider distribution of HL scores.

The psychometric properties of the Swedish version of the HLS-EU-Q16 questionnaire [18] were analysed according to the following criteria for measurement properties [27, 28]: internal consistency, reliability, construct validity, floor and ceiling effects, reproducibility (agreement), ease of scoring, time to administer and content validity.

### Internal consistency and reliability

Regarding internal consistency [27], in the first step of testing the original model of HLS-EU-Q-16 with the three domains of health care, disease prevention and health promotion [7] was examined using EFA [29]. Extraction in EFA was chosen to be based on Eigenvalue > 1. EFA was performed using the principal axis factoring extraction method, considered as relevant in our set of data, as it does not require normally distributed data [30]. Oblique Promax rotation method was used as it permits correlation of the factors [31]. Factor loadings with a cut-off of ≥.30 were included in the factors [32]. Different limits for communalities are found in the literature (0.2, 0.4 and 0.5) [33–35]. We have reported communalities irrespective of earlier suggested limits.

Since extracted four factors in the first step did not show the pattern of the original model of HLS-EU-Q16 [6] and a visual inspection of the scree plot in the first step indicated one factor solution, this was conducted in the second step. The extraction in the second step of EFA was forced to one fixed factor, otherwise the analysis was the same as in the first step.

Cronbach’s alpha was used to measure reliability and a level of > 0.80 was regarded as satisfactory [36].

### Construct validity

When testing construct validity [27], analyses of parental HL levels were conducted to find out which sub-groups had higher or lower levels of HL based on previous research. The participants (*N* = 190) were categorised into three groups based on their total HL scores (16–64); low HL (quartile 1: 29–45.0), middle HL (quartile 2: and quartile 3: 45.1–49.9) and high HL (quartile 4: 50–64). These categories were based on HL scores in relation to sociodemographic factors (parental age, region of birth, time of residence in Sweden, need for an interpreter, education) and variables about how parents perceived their ‘access to social support’ and ‘parental and child health’ showed whether HL in relation to these variables could be replicated in our study population, in line with previous research. Non-parametric tests, Kruskal-Wallis, and chi-square (×^2^) tests were used to calculate the relationships between the levels of HL and the aforementioned factors. A post hoc for Kruskal-Wallis was conducted with pairwise comparisons while the chi-square independence test was performed to test the differences between groups with Bonferroni correction for multiple tests.

### Floor and ceiling effects

Floor and ceiling effects were checked with a limit of less than 15% of the highest or lowest HL scores in the sample [28].

### Reproducibility: agreement

As HL in this study was measured only once, reproducibility: agreement [27] was evaluated through split-half testing for the HL measurement. The Spearman-Brown coefficient > 0.80 indicates a strong (reproducibility agreement) reliability for the instrument [37].

### Ease of scoring and time to administer

For a description of ease of scoring (the extent to which the measure can be scored by a trained investigator or expert) [27], the process of calculating total HL scores for the HLS-EU-Q instrument was reviewed. In our study, time to administer referred to duration of completing the entire structured interview including HLS-EU-Q16.

### Content validity

HLS-EU-Q instruments have in the development phase been evaluated by an group of experts in the field [5]. However, to discover more about content validity [27] in terms of face validity [38] for HLS-EU-Q16 (Swedish version) in this specific target group, the participants were encouraged to comment on the questions included in HLS-EU-Q16. This was performed to study how they perceived the content and to test the ease of use of the instrument. The interviewers noted the participants’ comments on HLS-EU-Q16 to find an overarching theme regarding their understanding of the items in the questionnaire.

## Results

### Descriptive data for sociodemographic background factors

The sociodemographic background factors of the participants are described in Table 1. More than 50% of the included infants were boys. Most of the participants were women and most of the participants were married/co-habiting with the other parent. The maternal mean age of the participants was less than 30 years, and the paternal mean age was closer to 35 years. Majority of the parents were born in Africa, followed by Sweden and the Middle East. More than 25% of the participants needed an interpreter for the interviews. The mean time of residence in Sweden for participants was around 13 years. The mean length of the education was around 13 years.

**Table 1:** Distribution of the sociodemographic factors of the participants (*N* = 164)

Infant’s gender % (*N* = 132)	
Girls	46.2 (61)
Boys	53.8 (71)
Parent’s gender % (*N* = 164)	
Women	74.4 (122)
Men	25.6 (42)
Marital status % (*N* = 164)	
Married/co-habiting	87.2 (143)
Married – living apart	6.7 (11)
Boyfriend/girlfriend – living apart – single	6.1 (10)
Parental age – Mean (SD) Range	30.2 (6.8) 17–64
Women – Mean (SD) Range	28.6 (5.5) 17–44
Men – Mean (SD) Range	34.9 (8.2) 24–64
Parents’ country/region of birth - % (*N* = 164)	
Sweden	26.8 (44)
Europe	8.5 (14)
Middle East (common MENA countries + Turkey)	20.7 (34)
Africa	31.1 (51)
Asia	12.2 (20)
North America/South America	*
Need of interpreter - % (*N* = 164)	
All parents	26.8 (44)
Women	28.7 (35)
Men	21.4 (9)
Years in Sweden (all parents) – Mean (SD) Range	12.9 (11.0) 0.1–39
Years in Sweden (women) – Mean (SD) Range	12.0 (11.7) 0.1–39
Years in Sweden (men) – Mean (SD) Range	15.5 (12.2) 1–37
Education (yrs.) (all parents) - Mean (SD) Range	12.9 (4.3) 0–21.5
Education (yrs.) (women) – Mean (SD)Range	12.9 (4.5) 0–20
Education (yrs.) (men) – Mean (SD) Range	12.9 (3.9) 2–21.5

*Not reported as *N* < 5

### Psychometric properties

#### Internal consistency and reliability

In the initial EFA, four factors with eigenvalues > 1 were extruded from the data set. The findings of the factor loadings (Additional file 3: Table 1) did not support the original subscales for HLS-EU-Q-16 with seven items in the health care domain, five items in disease prevention and four items in health promotion [7].

**Fig. 2:**
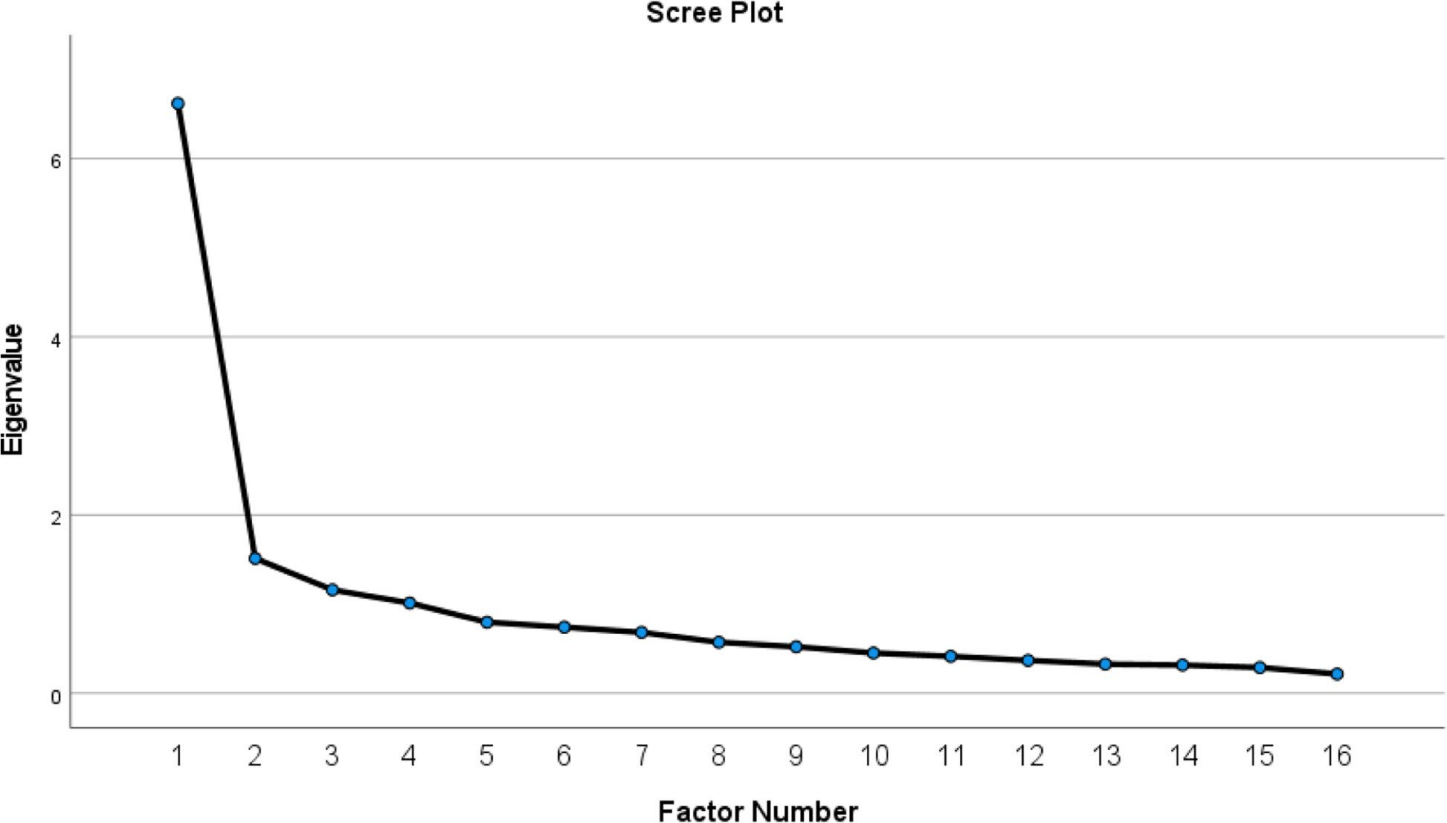
Scree plot of the initial EFA analysis, extracted four factors with Eigenvalue > 1

An inspection of the scree plot (Fig. 2) gave an indication to test one factor solution for the HLS-EU-Q16 scale. An EFA with one forced factor showed that the solution explained 37.7% of the total variance (Table 2). The approximate chi-square values suggested that correlations between the included items were sufficiently large for EFA (Table 2). All the items loaded at >.30, which was the cut-off point and are reported in detail together with initial and extracted communalities in Table 2. Bartlett’s test of sphericity was significant x^2^(120) = 1156.796 (*p* < .001) [34]. The Kaiser-Meyer-Olkin (KMO) value (Table 2) indicated that the data were suitable for data analyses [39].

**Table 2:** Factor loading and communalities (both initial and extracted) from EFA for one factor solution, eigenvalue and extracted % of variance, KMO and reliability analysis (Cronbach’s α)

Items	Factor loadings	Communalities	
		initial	extracted
j) How easy/difficult is it for you to understand why you need health screenings (such as breast examinations, blood sugar or blood pressure tests)? *Disease prevention*	.779	.640	.607
g) How easy/difficult is it for you to follow instructions from your doctor or pharmacist? *Health care*	.708	.572	.501
d) How easy/difficult is it for you to understand your doctor’s or pharmacist’s instructions on how to take a prescribed medicine? *Health care*	.703	.588	.494
i) How easy/difficult is it for you to understand warnings about behaviours (e.g., smoking, low physical activity and drinking too much)? *Disease prevention*	.701	.502	.492
b) How easy/difficult is it for you to find out where to get professional help when you are ill (e.g., doctor, pharmacist, or psychologist)? *Health care*	.697	.606	.486
o) How easy/difficult is it for you to understand information in the media on how to get heathier (e.g., from the internet, daily or weekly magazines? *Health promotion*	.694	.524	.482
m) How easy/difficult is it for you to find out about activities that are good for your mental well-being (e.g., meditation, exercise, and walking)? *Health promotion*	.667	.465	.445
c) How easy/difficult is it for you to understand what your doctor says to you? *Health care*	.664	.492	.440
f) How easy/difficult is it for you to use the information your doctor gives you to make decisions about your illness? *Health care*	.655	.542	.429
a) How easy/difficult is it for you to find information about treating illnesses that concern you? *Health care*	.615	.464	.378
p) How easy/difficult is it for you to judge what every day behaviours are related to your health (e.g., eating habits, exercise habits and drinking habits)? *Health promotion*	.512	.349	.262
h) How easy/difficult is it for you to find information on how to manage mental health problems such as stress and depression? *Disease prevention*	.496	.316	.246
l) How easy/difficult is it for you to decide how you can protect yourself from illness based on information in the media (e.g., newspapers, leaflets, and the Internet)? *Disease prevention*	.481	.528	.232
e) How easy/difficult is it for you to judge when you need to get a second opinion from another doctor? *Health care*	.460	.339	.211
k) How easy/difficult is it for you to judge whether the information on health risks in the media is reliable (e.g., TV or the Internet)? *Disease prevention*	.424	.447	.179
n) How easy/difficult is it for you to understand advice on health from your family members or friends? *Health promotion*	.401	.351	.160
Eigenvalue (extracted)	6.044		
Extracted % of variance	37.774		
Kaiser-Meyer-Olkin Measure of Sampling Adequacy	.892		
Cronbach’s α	.900		

The reliability measure obtained using Cronbach’s alpha was satisfactory (Table 2). The exclusion of items did not strengthen the reliability.

### Construct validity

The results from the analyses of relationships between HL levels and sociodemographic background factors (parental age, region of birth, years in Sweden, need for an interpreter and education), as well as HL levels in relation to access to social support and parental and child health as perceived by the participants in the interviews are presented in Table 3.

**Table 3:** Distribution of sociodemographic factors, access to social support and parental and child health in relation to low, middle, and high levels of HL

Sociodemographic Characteristics	Low HL	Middle HL	High HL	Kruskal-Wallis/X^2^
Parental age (Mean)	30.5	31.0	28.8	2.084 *p* = .353
Country/region of birth (%)				52.339 *p* < .001
Sweden	6.8	25.0	68.2	
Europe	50.0	42.9	7.1	
Middle East	35.3	55.9	8.8	
Africa	37.3	39.2	23.5	
Asia	50.0	40.0	10.0	
North America/South America*	*	*	*	
Years in Sweden (Mean)	7.4	10.7	21.6	36.405 *p* < .001
Need for an interpreter (%)				24.157 *p* < .001
Interpreter	52.3	45.5	2.3	
No need for an interpreter	33.3	37.5	39.2	
Education (yrs.) (Mean)	11.1	13.1	14.7	15.844 *p* < .001
Access to social support (%)				11.895 *p* = .018
Always	30.5	35.6	33.9	
Most of the time	37.5	37.5	25.0	
Never/mostly not	21.4	78.6	0.0	
Parental health (%)				5.473 *p* = .242
Excellent/good	28.7	41.2	30.1	
Average	35.7	39.3	25.0	
Poor/Very poor	*	*	*	
Child health (%)				1.9036 *p* = .386
Excellent/good	31.6	38.6	29.7	
Average	16.7	66.7	16.7	
Poor/very poor	*	*	*	

*Not displayed as *N* < 5

There were no statistically significant differences in levels of HL by age, but between the parents’ region of birth. The chi-square independence test with Bonferroni correction for multiple tests, indicated (*p* = .05) that Swedish-born parents had significantly higher HL compared to parents from all the other geographical regions.

Concerning HL in relation to years of residence in Sweden, pairwise comparisons between the groups indicated that parents with longer residency in Sweden had significantly higher HL. Significant differences were observed between groups having a high and low HL (*p* < .001) and high and middle HL (*p* < .001) but not between middle and low HL (*p* = .170).

Similarly, statistically significant differences in HL were observed between parents who needed an interpreter and those who did not need one. Chi-square independence test with Bonferroni correction for multiple tests (*p* = .05) that parents who needed an interpreter had a lower level of HL compared to those who did not need one.

Furthermore, statistically significant findings were obtained concerning HL and years of education. Pairwise comparisons between the groups indicated that parents with a high HL had higher level of education compared to parents with middle HL (*p* = .0130) and parents with low HL (*p* < .000).

Regarding access to social support, statistically significant association was observed between HL levels and access to support. Chi-square independence test with Bonferroni correction for multiple tests showed (*p* = .05) that parents who always had access to support had significantly higher levels of HL compared with parents who mostly not/never have access to support. No statistically significant associations were found between levels of HL and parental health or child health.

### Floor and ceiling effects

The lowest obtained total score in this study was 29 and the highest 64. None of the participants scored the lowest possible total score of HL (16 scores). Only a few participants (*N* = 4, 2.4% scored the highest possible score (64 scores).

### Reproducibility: agreement

The Spearman-Brown coefficient of split-half testing, 0.836, indicate strong agreement.

### Ease of scoring and time to administer

Calculation of the total scores for HLS-EU-Q16 was assessed either gained by adding 16 items, each of them giving points of 1-4 (total 16–64), or by dichotomising according to the described procedures (total 0–16) [7]. The interview duration was measured for 23 interviews and was 11–30 min, including all the questionnaire questions, with an average of 17.5 min.

### Content validity

The participants made several comments about the questions included in HLS-EU-Q16 regarding how they understood and perceived the questions. The participants who had grown up in Sweden primarily regarded the questions in HLS-EU-Q16 as easy and understandable. However, they regarded some of the questions as being rather complex, requiring them to think before responding. Participants with a mother tongue other than Swedish/English who did not need an interpreter indicated that the questions were advanced or lacking clarity without further explanation. They regarded some of the questions as being easier and more straightforward. For example, question *g – How easy/difficult is it for you to follow instructions from your doctor or pharmacist?* Whereas, for example, question *e – How easy/difficult is it for you to judge when you need to get a second opinion from another doctor?* was regarded as complex and even difficult to understand.

The participants who used an interpreter for the interview stated that their inability to speak Swedish as their main problem regarding health-related issues and the HLS-EU-Q16 questionnaire. This was also evident in answers to, for example, the question about the ease of understanding what their doctor says (see question *c* in Table 2). A recurring theme was that if an interpreter was present, it was easy to understand what the doctor says.

Parents who worked in the healthcare sector as medical doctors, pharmacists, nurses, and assistant nurses stated that questions in HLS-EU-Q16 were familiar and easy to understand, regardless of whether they were born in Sweden.

## Discussion

The results of this study, aimed at gaining knowledge about the psychometric properties of HLS-EU-Q16 (Swedish version) [18], demonstrated acceptable psychometric qualities for the instrument when measuring comprehensive HL among first-time parents living in multicultural settings in Sweden and confirmed one factor solution for the instrument. Our findings did not support the proposed original model of HLS-EU-Q, which measures HL in the three domains of health care, disease prevention and health promotion [5, 7].

The analysis of internal consistency, through an exploratory factor analysis, confirmed one factor solution in HLS-EU-Q16, explaining 37.7% of the total variance. The finding of high reliability is in line with the results from the validation process of HLS-EU-Q16 (English version) [6, 7].

The construct validity of HLS-EU-Q16 was approved by findings with the study population categorised into three groups based on their total HL scores (low HL, middle HL, high HL). These findings showed that parents born outside Sweden, those who had lived for a shorter time in Sweden and those with poorer Swedish language proficiency, as well as parents with a lower level of education demonstrated significantly lower levels of HL, in line with previous research [1, 8, 11, 13]. Furthermore, participants always having access to support from social networks had significantly higher HL compared with those who never/most of the time not had access to support. Even this finding is in line with previous studies [13].

No floor effect of total HL scores was found in our sample and the ceiling effect was low. However, there was a tendency among participants to score high rather than low in HL. The lowest total score for participants was 29, which is much higher than the lowest possible (16 scores). Furthermore, a pre-analyses of HL that applied dichotomisation showed the same tendency among participants to score high, as more than 60% of the participants would have ended up in the category of ‘likely sufficient HL’ [7]. A social-desirability bias might be present in the data collection [40], meaning that the participants may have tended to answer HLS-EU-Q16 questions in a manner that was viewed as favourable for the interviewers in the face-to-face interviews. The results from a previous study show that parents tend to score high, for example, in questions about parental self-efficacy when interviewed in person [23]. Regarding reproducibility: agreement of the instrument, the reliability of the instrument was good. Concerning ease of scoring, the process of calculating total scores for HLS-EU-Q16 was smooth. Time to administer was mainly not recorded for the interviews or HLS-EU-Q16, which is a clear limitation of this study.

In the analyses of content validity, the interpreters and participants sometimes had a limited understanding of the HLS-EU-Q16 questions, which appeared to be one of the greatest challenges and limitations of this study. While the Swedish-born participants primarily regarded the HLS-EU-16 questions as being easy and understandable, the participants born abroad, with a mother tongue other than Swedish/English, stated that the HLS-EU-Q16 questions were advanced and lacking clarity without further explanation. The participants who needed an interpreter stated that their difficulty with health-related issues was generally due to their lack of Swedish language proficiency. However, regardless of their country of birth, parents working in the Swedish healthcare sector stated that HLS-EU-Q16 included familiar questions, in line with previous findings [15].

The previous development of the original HLS-EU-Q instrument for measuring comprehensive HL in different populations has clear aims and involved experts of the field [5] which support the content validity of the HLS-EU-Q16 instrument. The clear-cut aim of measuring comprehensive HL using HLS-EU-Q16 (Swedish version) among first-time parents with infants in the multicultural setting presented in this study gave further input to the content validity.

Even if the findings indicated that HLS-EU-Q16 is a valid instrument for the multicultural settings being studied, ‘variance explained’ for the one factor solution in this study was not as high as a general rule 50% that is recommended in the literature [41]. Few low communalities were found in the one factor solution in this study if compared to the lowest advisable limit for communalities (>.2) [35]. Our data set had a relatively high number of multivariate outliers (*N* = 26) that were removed from the data set. Higher sample size may be needed for future studies in similar settings. However, the alternative statistical analyses that were conducted without removal of multivariate outliers (*N* = 190) (Additional file 2) did only slightly deviate from the results reported here.

As already mentioned, challenges were found related to how well the HLS-EU-Q16 questions were understood by the target population. It is common for migrants to be excluded from research that requires interpreters because of the methodological issues [42], while minority groups are very often willing to participate [43], as they were in this study. Methodologically, the inclusion of participants with poor Swedish language proficiency and the use of interpreters for the interviews, as well as translations of the HLS-EU-Q16 instrument as support for interpreters, could be questioned in our study. However, the reality of the multicultural settings makes it difficult to strictly adhere to methodological procedures. Exclusion of participants with poorer Swedish language proficiency and those who need a translator or prefer to be interviewed in English, would have given much lower participation rate than the initial 73.1% that was achieved in this study. Furthermore, we would have missed the knowledge of how HLS-EU-Q16 is perceived by migrants that constitute the majority in our sample and the target group of our study.

Our decision to include participants from a migrant background highlighted the reality that non-Swedish speakers struggle with readability and comprehension of the HLS-EU-Q16 questions in this study. Furthermore, it also highlights the wider challenges that migrants face with regards to not just understanding health-related research interview questions, but also health-related knowledge, information, healthcare systems, etc. in their new countries. These challenges are well-known and several barriers including low HL have been identified among migrants in Sweden, such as avoidance of health care because of poor language proficiency, doubts about not receiving the necessary help, long waiting times or lacking knowledge about how to access health care [17]. Our findings are in line with previous studies showing that migrants generally score lower on HL measures compared to majority populations [8, 17] as we found that parents who scored a low level of HL were foreign-born, had shorter residency in Sweden, as well as parents who needed an interpreter because of their poor Swedish language proficiency. From a child health perspective, it is important to address parental HL in multicultural settings as there is evidence that parents’ low HL can negatively affect their child’s health in several ways and it is known that parents play a key role in providing and promoting the health of their children [44]. Interventions that support parental HL in multicultural settings are very necessary.

In line with previous studies, this study found that a higher educational level is associated with a high level of HL [1, 8, 11]. This study did not specifically analyse whether there were differences in HL levels between those who were educated in Sweden or in other educational settings outside Sweden. What is known from a previous research review is that educational achievement is one of the most important determinants of HL [11].

Furthermore, a significant association in our findings regarding access to support from social network, could indicate what has been seen in previous studies, that receiving social support can be associated with a higher level of HL [13]. The importance of social support has been highlighted in a previous study suggesting that HL research should focus beyond individual HL as the HL scores of individuals and their family members can be positively correlated [14]. However, in multicultural settings, access to social networks can vary from migrating alone, leaving the network behind, to having access to a larger social network in Sweden that provides social support. Further studies regarding the association between HL and social support in multicultural settings could be useful for increasing the understanding of how the different social networks of individuals could support their HL.

Finally, it is suggested that studies are conducted that measure parental HL in multicultural settings and in relation to child health to identify better and more effective ways of improving parental HL that has additional benefits on child health. Qualitative interviews could be a useful component for finding deeper explanations of parental HL levels in relation to child health in multicultural settings.

## Conclusions

The Swedish version of HLS-EU-Q16 could be used together with other instruments for measuring the overall HL in multicultural settings, despite the language-related challenges. HLS-EU-Q16 appears to discriminate between different levels of HL in relation to different sociodemographic factors such as migrant background (persons born outside Sweden, who have spent less time in Sweden and poorer Swedish language proficiency), shorter education and limited access to support from social networks. However, other measures of HL which should be adapted to use in multicultural settings, need to be explored in further studies of parental HL and its relationship to child health in multicultural settings.

## Supplementary Information

**Additional file 1: Table 1.** Univariate statistics for the items a-p in HLS-EU-Q16. **Table 2.** Skewness and kurtosis of each item as well as results from Kolmogorov-Smirnov’s test of normality for each item in HLS-EU-Q16 before replacing missing values with series means. **Table 3.** Mardia’s multivariate normality test. Sample size: 190. Number of variables: 16.

**Additional file 2: Table 1.** Distribution of the sociodemographic factors of the participants, *N* = 190. **Table 2.** Factor loading and communalities (both initial and extracted) from EFA for one factor solution, eigenvalue and extracted % of variance, KMO and reliability analysis (Cronbach’s α). *N* = 190. **Table 3.** Distribution of sociodemographic factors, access to social support and parental and child health in relation to low, middle, and high levels of HL, *N* = 190.

**Additional file 3: Table 1.** The initial EFA analysis of HLS-EU-Q16, extracted four factors with Eigenvalue > 1. Factor loadings.

## Data Availability

The datasets generated and analysed during the current study are not publicly available due to the ethical approval (registration number 2017/1587-31/5) which states that the use of interview data should respect anonymity of participants, only be accessed as whole by the research group, and be stored locked by passwords. Data are available from the corresponding author upon a reasonable request.

## Abbreviations

CHC: Child healthcare
EFA: Exploratory factor analyses
HL: Health Literacy
HLS-EU-Q: European Health Literacy Survey Questionnaire
KMO: Kaiser-Meyer-Olkin
