# Supplementary material for: Psychometric qualities of the HLS-EU-Q16 instrument for parental health literacy in Swedish multicultural settings
Source: BMC Public Health. 2022 Feb 12;22:293. doi: 10.1186/s12889-021-12346-8 (PMC8841050; doi:10.1186/s12889-021-12346-8)
Supplement: Supplementary file 1 — Additional file 1: Table 1. Univariate statistics for the items a-p in HLS-EU-Q16. Table 2. Skewness and kurtosis of each item as well as results from Kolmogorov-Smirnov’s test of normality for each item in HLS-EU-Q16 before replacing missing values with series means. Table 3. Mardia’s multivariate normality test. Sample size: 190. Number of variables: 16. [file 12889_2021_12346_MOESM1_ESM.docx]

Additional file 1, Table1. Univariate statistics for the items a-p in HLS-EU-Q16.

|  | **N** | **Mean** | **Std. Deviation** | **Missing** | | **No. of Extremes ^a.b^** | |
| --- | --- | --- | --- | --- | --- | --- | --- |
|  |  |  |  | **N** | **%** | **Low** | **High** |
| **HLS_a** | 188 | 3.02 | .716 | 2 | 1.1 | . | . |
| **HLS_b** | 189 | 2.92 | .694 | 1 | .5 | . | . |
| **HLS_c** | 188 | 3.16 | .620 | 2 | 1.1 | 2 | 0 |
| **HLS_d** | 190 | 3.23 | .581 | 0 | .0 | 0 | 0 |
| **HLS_e** | 184 | 2.62 | .633 | 6 | 3.2 | 0 | 0 |
| **HLS_f** | 187 | 2.83 | .577 | 3 | 1.6 | 0 | 0 |
| **HLS_g** | 190 | 3.07 | .519 | 0 | .0 | . | . |
| **HLS_h** | 182 | 2.76 | .654 | 8 | 4.2 | 0 | 0 |
| **HLS_i** | 190 | 3.12 | .610 | 0 | .0 | . | . |
| **HLS_j** | 190 | 3.08 | .628 | 0 | .0 | . | . |
| **HLS_k** | 190 | 2.65 | .703 | 0 | .0 | 0 | 0 |
| **HLS_l** | 188 | 2.79 | .598 | 2 | 1.1 | 0 | 0 |
| **HLS_m** | 188 | 2.98 | .589 | 2 | 1.1 | . | . |
| **HLS_n** | 189 | 3.23 | .552 | 1 | .5 | 1 | 0 |
| **HLS_o** | 188 | 2.93 | .607 | 2 | 1.1 | . | . |
| **HLS_p** | 190 | 3.14 | .619 | 0 | .0 | 2 | 0 |
| a. Number of cases outside the range (Q1 - 1.5*IQR. Q3 + 1.5*IQR). | | | | | | | |
| b. . indicates that the inter-quartile range (IQR) is zero. | | | | | | | |

Additional file 1, table 2. Skewness and kurtosis of each item as well as results from Kolmogorov-Smirnov’s test of normality for each item in HLS-EU-Q16 before replacing missing values with series means.

|  | Skewness | Kurtosis | Kolmogorov-Smirnov |
| --- | --- | --- | --- |
| HLS_a | -.561 | .540 | >.001 |
| HLS_b | -.560 | .738 | >.001 |
| HLS_c | -.395 | .711 | >.001 |
| HLS_d | -.073 | -.393 | >.001 |
| HLS_e | -.133 | -.131 | >.001 |
| HLS_f | -.153 | .218 | >.001 |
| HLS_g | .101 | .673 | >.001 |
| HLS_h | -.306 | .246 | >.001 |
| HLS_i | -.209 | .239 | >.001 |
| HLS_j | -.193 | .027 | >.001 |
| HLS_k | -.214 | -. 076 | >.001 |
| HLS_l | -.194 | .186 | >.001 |
| HLS_m | -.631 | 2. 049 | >.001 |
| HLS_n | -.137 | .783 | >.001 |
| HLS_o | -.109 | .133 | >.001 |
| HLS_p | -.366 | .695 | >.001 |

Additional file 1, table 3. Mardia’s multivariate normality test. Sample size: 190. Number of variables: 16.

|  | b | z | p-value |
| --- | --- | --- | --- |
| Skewness | 64.17436 | 2032.1881 | 0 |
| Kurtosis | 363.63699 | 21.7205 | 0 |
