# Supplementary material for: Psychometric qualities of the HLS-EU-Q16 instrument for parental health literacy in Swedish multicultural settings
Source: BMC Public Health. 2022 Feb 12;22:293. doi: 10.1186/s12889-021-12346-8 (PMC8841050; doi:10.1186/s12889-021-12346-8)
Supplement: Supplementary file 2 — Additional file 2: Table 1. Distribution of the sociodemographic factors of the participants, N = 190. Table 2. Factor loading and communalities (both initial and extracted) from EFA for one factor solution, eigenvalue and extracted % of variance, KMO and reliability analysis (Cronbach’s α). N = 190. Table 3. Distribution of sociodemographic factors, access to social support and parental and child health in relation to low, middle, and high levels of HL, N = 190. [file 12889_2021_12346_MOESM2_ESM.docx]

Additional file 2. Results from data analyses N=190.

Additional file 2, table 1. Distribution of the sociodemographic factors of the participants, N=190.

| Infant’s gender % (N =156)  Girls  Boys | 46.8 (73)  53.2 (83) |
| --- | --- |
| Parent’s gender % (N=190)  Women  Men | 74.7 (142)  25.3 (48) |
| Marital status % (N=190)  Married/co-habiting  Married – living apart  Boyfriend/girlfriend – living apart – single | 85.8 (163)  6.3 (12)  7.9 (15) |
| Parental age – Mean (SD) Range  Women – Mean (SD) Range  Men – Mean (SD) Range | 30.1 (6.7) 17–64  28.6 (5.5) 17–44  34.8 (7.9) 24–64 |
| Parents’ country/region of birth - % (N=190)  Sweden  Europe  Middle East (common MENA countries + Turkey)  Africa  Asia  North America/South America | 24.7 (47)  8.9 (17)  19.5 (37)  34.2 (65)  12.1 (23)  * |
| Need of interpreter - % (N=193)  All parents  Women  Men | 25.3 (48)  27.5 (39)  18.8 (9) |
| Years in Sweden (all parents) – Mean (SD) Range  Years in Sweden (women) – Mean (SD) Range  Years in Sweden (men) – Mean (SD) Range | 12.4 (11.6) 0.1–39  11.5 (11.4) 0.1–39  15.0 (11.9) 1–37 |
| Education (yrs.) (all parents) - Mean (SD) Range  Education (yrs.) (women) – Mean (SD)Range  Education (yrs.) (men) – Mean (SD) Range | 12.9 (4.1) 0–21.5  12.8 (4.3) 0–20  13.1 (3.8) 2–21.5 |

*Not reported as N<5.

Additional file 2, table 2. Factor loading and communalities (both initial and extracted) from EFA for one factor solution, eigenvalue and extracted % of variance, KMO and reliability analysis (Cronbach’s α). N=190.

| Items | Factor loadings | Communalities | |
| --- | --- | --- | --- |
|  |  | initial | extracted |
| j) How easy/difficult is it for you to understand why you need health screenings (such as breast examinations, blood sugar or blood pressure tests)? *Disease prevention* | .779 | .641 | .607 |
| g) How easy/difficult is it for you to follow instructions from your doctor or pharmacist? *Health care* | .707 | .572 | .500 |
| i) How easy/difficult is it for you to understand warnings about behaviours (e.g., smoking, low physical activity and drinking too much)? *Disease prevention* | .699 | .503 | .489 |
| b) How easy/difficult is it for you to find out where to get professional help when you are ill (e.g., doctor, pharmacist, or psychologist)? *Health care* | .697 | .606 | .485 |
| o) How easy/difficult is it for you to understand information in the media on how to get heathier (e.g., from the internet, daily or weekly magazines? *Health promotion* | .694 | .524 | .481 |
| d) How easy/difficult is it for you to understand your doctor’s or pharmacist’s instructions on how to take a prescribed medicine? *Health care* | .692 | .585 | .478 |
| m) How easy/difficult is it for you to find out about activities that are good for your mental well-being (e.g., meditation, exercise, and walking)? *Health promotion* | .668 | .464 | .446 |
| c) How easy/difficult is it for you to understand what your doctor says to you? *Health care* | .660 | .497 | .435 |
| f) How easy/difficult is it for you to use the information your doctor gives you to make decisions about your illness? *Health care* | .648 | .545 | .420 |
| a) How easy/difficult is it for you to find information about treating illnesses that concern you? *Health care* | .616 | .464 | .379 |
| p) How easy/difficult is it for you to judge what every day behaviours are related to your health (e.g., eating habits, exercise habits and drinking habits)? *Health promotion* | .510 | .354 | .260 |
| h) How easy/difficult is it for you to find information on how to manage mental health problems such as stress and depression? *Disease prevention* | .488 | .327 | .238 |
| l) How easy/difficult is it for you to decide how you can protect yourself from illness based on information in the media (e.g., newspapers, leaflets, and the Internet)? *Disease prevention* | .472 | .523 | .223 |
| e) How easy/difficult is it for you to judge when you need to get a second opinion from another doctor? *Health care* | .441 | .342 | .194 |
| k) How easy/difficult is it for you to judge whether the information on health risks in the media is reliable (e.g., TV or the Internet)? *Disease prevention* | .412 | .444 | .170 |
| n) How easy/difficult is it for you to understand advice on health from your family members or friends? *Health promotion* | .395 | .352 | .156 |
| Eigenvalue (extracted) | 5.962 | | |
| Extracted % of variance | 37.265 | | |
| Kaiser-Meyer-Olkin Measure of Sampling Adequacy | .888 | | |
| Cronbach’s α | .898 | | |

Additional file 2, table 3. Distribution of sociodemographic factors, access to social support and parental and child health in relation to low, middle, and high levels of HL, N=190.

| Sociodemographic Characteristics | Low HL | Middle HL | High HL | Kruskal-Wallis/X^2^ |
| --- | --- | --- | --- | --- |
| Parental age (Mean) | 30.5 | 30.6 | 28.8 | 1.723 p=.422 |
| Country/region of birth (%)  Sweden  Europe  Middle East  Africa  Asia  North America/South America* | 6.4  41.2  32.4  29.2  43.5  * | 29.8  52.9  59.5  52.3  47.8  * | 63.8  5.9  8.1  18.5  8.7  * | 55.463 p<.001 |
| Years in Sweden (Mean) | 7.4 | 10.3 | 21.6 | 38.138 p<.001 |
| Need for an interpreter (%)  Interpreter  No need for an interpreter | 47.9  19.7 | 50.0  47.2 | 2.1  33.1 | 24.346 p<.001 |
| Education (yrs.) (Mean) | 11.1 | 13.0 | 14.7 | 17.347 p<.001 |
| Access to social support (%)  Always  Most of the time  Never/mostly not | 25.9  33.3  20.0 | 45.3  44.4  80.0 | 28.8  22.2  0.0 | 8.983 p=.062 |
| Parental health (%)  Excellent/good  Average  Poor/Very poor | 24.4  35.7  * | 50.0  39.3  * | 25.6  25.0  * | 7.225 p=.124 |
| Child health (%)  Excellent/good  Average  Poor/very poor | 27.1  16.7  * | 57.3  66.7  * | 25.5  16.7  * | 0.876 p=.645 |

*Not displayed as N<5.
