# Supplementary material for: Psychometric qualities of the HLS-EU-Q16 instrument for parental health literacy in Swedish multicultural settings
Source: BMC Public Health. 2022 Feb 12;22:293. doi: 10.1186/s12889-021-12346-8 (PMC8841050; doi:10.1186/s12889-021-12346-8)
Supplement: Supplementary file 3 — Additional file 3: Table 1. The initial EFA analysis of HLS-EU-Q16, extracted four factors with Eigenvalue > 1. Factor loadings. [file 12889_2021_12346_MOESM3_ESM.docx]

Additional file 3, table 1. The initial EFA analysis of HLS-EU-Q16, extracted four factors with Eigenvalue >1. Factor loadings.

| Items | Domain | Factor 1 | Factor 2 | Factor 3 | Factor 4 |
| --- | --- | --- | --- | --- | --- |
|  |  |  |  |  |  |
| j) How easy/difficult is it for you to understand why you need health screenings (such as breast exam, blood sugar- or blood pressure test? | (*Understand disease prevention)* | .806 | .636 | .316 | .332 |
| g) How easy/difficult is it for you to follow instructions from your doctor or pharmacist? | (*apply/use information health care)* | .769 | .554 | .282 | .260 |
| d) How easy/difficult is it for you to understand your doctor’s or pharmacist’s instruction on how to take a prescribed medicine? | *(Understand information health care)* | .748 | .541 | .383 | .216 |
| c) How easy/difficult is it for you to understand what your doctor says to you? | (*Understand information – health care)* | .734 | .480 | .288 | .300 |
| i)How easy/difficult is it for you to understand warnings about behavior (e.g., smoking, low physical activity and drinking too much)? | (*Understand information disease prevention)* | .709 | .573 | .374 | .236 |
| m) How easy/difficult is it for you to find out about activities that are good for your mental well-being (e.g. meditation, exercise and walking)? | (*Obtain/access information*  *Health promotion* | .595 | .585 | .395 | .369 |
| e) How easy/difficult is it for you to judge when you need to get a second opinion from another doctor? | *(Process/apprise information health care)* | .494 | .480 | .316 | -.213 |
| b) How easy/difficult is it for you to find out where to get professional help when you are ill (e.g., doctor, pharmacist, or psychologist)? | (*Obtain access information health care)* | .575 | .840 | .282 | .253 |
| a) How easy/difficult is it for you to find information on treatments of illness that concerns you? | (*Obtain/access information health care)* | .507 | .688 | .250 | .279 |
| f) How easy/difficult is it for you to use information the doctor gives you to make decision about your illness? | (*Apply/use information health care*) | .652 | .679 | .348 | -.041 |
| o) How easy/difficult is it for you to understand information in the media on how to get heathier (e.g., from the internet, daily or weekly magazines? | (*Understand information health promotion)* | .562 | .671 | .438 | .460 |
| h) How easy/difficult is it for you to find information on how to manage mental health problems such as stress and depression? | (*Obtain/access information disease prevention)* | .398 | .552 | .316 | .135 |
| l) How easy/difficult is it for you to decide how you can protect yourself from illness based on information in media (e.g., newspapers, leaflets, and Internet)? | (*Apply /use information disease prevention)* | .406 | .344 | .988 | .289 |
| k) How easy/difficult is it for you to judge if the information on health risks in media is reliable (e.g., TV or Internet)? | *(Process/apply information disease prevention)* | .277 | .388 | .615 | .342 |
| n) How easy/difficult is it for you to understand advice on health from your family members or friends? | (*Understand information health promotion)* | .319 | .275 | .346 | .589 |
| p) How easy/difficult is it for you to judge which everyday behaviors is related to your health (e.g., eating habits, exercise habits and drinking habits)? | *(Process/appraise information health promotion)* | .438 | .426 | .288 | .481 |
